# Comparison of Anatomical and Nonanatomical Hepatectomy for Colorectal Liver Metastasis: A Meta-Analysis of 5207 Patients

**DOI:** 10.1038/srep32304

**Published:** 2016-08-31

**Authors:** Haowen Tang, Bingmin Li, Haoyun Zhang, Jiahong Dong, Wenping Lu

**Affiliations:** 1 Hospital and Institute of Hepatobiliary Surgery, Chinese PLA General Hospital, 28 Fuxing Road, Haidian, Beijing, 100853, China; 2 Medical School of Chinese PLA, 28 Fuxing Road, Haidian, Beijing, 100853, China; 3 Center for Hepatopancreatobiliary Diseases, Beijing Tsinghua Changgung Hospital, Tsinghua University Medical Center, 168 Litang Road, Changping, Beijing, 102218, China

## Abstract

It remains unclear whether hepatectomy for colorectal liver metastasis (CRLM) should be performed as anatomical resection (AR) or nonanatomical resection (NAR). The aim of this study is to compare the short- and long-term outcomes of AR and NAR for CRLM. PubMed, Web of Science, EMBASE and the Cochrane Library were systematically searched to identify eligible studies. Twenty one studies involving 5207 patients were analyzed: 3034 (58.3%) underwent AR procedure and 2173 (41.7%) underwent NAR procedure. The results showed that overall survival (OS, hazard ratio (HR) 1.06, 95% confidence interval (CI) 0.95–1.18) and disease free survival (DFS, HR 1.11, 95% CI 0.99–1.24) did not differ significantly between AR and NAR. Duration of operation, postoperative morbidity and mortality were higher in AR than in NAR. There were no significant differences in blood loss and prevalence rate of postoperative positive margins (OR 0.79, 95% CI 0.37–1.52). Our analysis shows that AR does not seem to bring more prognostic benefits than NAR for the treatment of CRLM, and does seem to be inferior to NAR in terms of duration of operation, incidence of postoperative morbidity and mortality.

Colorectal cancer (CRC) is one of the most common human malignancies. Worldwide, approximately 1.2 million new cases are diagnosed and over 600 thousand deaths are estimated to occur annually^1^. About 40% patients with CRC develop liver metastasis at the time of presentation, with approximately 20% presenting as synchronous metastasis (within 6 months of resection of the primary tumor) and the remaining 20% as metachronous metastasis (after this period)^2^,^3^,^4^. Liver resection has been shown to present the best chance of cure in the treatment of colorectal liver metastasis (CRLM), with a five-year survival rate exceeding 50% and nearly 20% postoperative patients surviving more than ten years^5^,^6^,^7^,^8^.

Anatomical resection (AR)^9^ for hepatocellular carcinoma (HCC), through a systematic removal of the liver parenchyma of one or more Couinaud’s segments fed by portal branches bearing the HCC, may reasonably reduce recurrence and return survival benefits compared to nonanatomical resection (NAR) or wedge resection. Through decades of practice, a widely accepted survival superiority of AR has been confirmed by several large cohort studies and meta-analyses^10^,^11^,^12^,^13^,^14^,^15^,^16^,^17^. Similar to HCC, in CRLM tumor cells from colorectal primary lesions also travel and spread via the portal vein as an afferent and efferent vessel^18^. However, whether an analogical survival superiority can be achieved by AR for CRLM in comparison with NAR remains unclear. Several reports^19^,^20^,^21^ have documented long-term survival benefits of AR procedure for CRLM over NAR procedure, whereas other reports^22^,^23^,^24^,^25^ have failed to demonstrate such benefits, with results showing equivalent five-year overall survival (OS) rate between the two procedures.

Therefore, a meta-analysis of all available studies comparing the efficacy (short- and long-term outcomes) of AR procedure and NAR procedure for CRLM was conducted to get more reliable and up-to-date evidence.

## Methods

The meta-analysis was conducted in adherence with the recommendations of the Preferred Reporting Items for Systematic Reviews and Meta-Analyses (PRISMA) guidelines^26^,^27^. All analyses were based on previously published studies, thus no ethical approval and patient consent are required. To ensure accuracy and minimize bias, all vital stages of the analysis were carried out separately by two reviewers; any disagreement was settled through consensus discussion.

### Study Selection

A systematic literature search of PubMed, Web of Science, EMBASE and the Cochrane Library was performed to select articles comparing AR with NAR for CRLM. Searches were limited to studies published in English from the initiation of the databases to June, 2016. No additional restrictions were applied to the searches with regard to region or publication type. The following medical subject headings (MeSH) were used: “Colorectal Neoplasms,” “Neoplasm Metastasis,” “Liver Neoplasms” and “Hepatectomy”. Besides, the following keywords were used to complete the literature search: “Hepatectomy,” “liver resection,” “hepatic resection,” “anatomic/anatomical,” “nonanatomic/ nonanatomical,” “major,” “minor,” “limited,” “wedge,” “CRLM/CLM” and “colorectal liver metastasis.” Furthermore, the references given in the retrieved papers were manually checked for further relevant articles. In the case of repeated studies describing the same group of population, only the most recent or the highest in quality was included. The latest search was performed on July 3, 2016. To ensure the reliability and verifiability of our analysis, eligible studies were identified in according with the following inclusion and exclusion criteria. Inclusion criteria were: (1) studies on human; (2) patients with pathologically confirmed diagnoses of CRLM primarily undergoing potentially curative resections; (3) articles comparing AR with NAR; (4) articles reporting short- and (or) long-term outcomes of AR and NAR. A study had to meet all four inclusion criteria for inclusion. Exclusion criteria were: (1) review articles, letters, case reports, editorials or comments and conference abstracts. (2) articles with no clear definition or grouping of AR and NAR. (3) main outcomes of interest not stated or impossible to calculate. (4) articles including patients mainly undergoing repeated hepatectomy for CRLM or with unresectable extrahepatic metastases. A study meeting any of the four exclusion criteria was excluded.

### Data Extraction and Definition

The following relevant parameters were extracted and summarized independently by two reviewers (Haowen Tang and Bingmin Li) for each study included in the meta-analysis: first author, study region, year of publication, total sample size, number of patients enrolled in each group (AR and NAR), population characteristics, primary tumor location, liver metastasis characteristics, short-term outcomes (operative and post-operative data) and long-term outcomes (OS and disease free survival (DFS)). At the same time, each screened article was graded by the Newcastle-Ottawa Scale (NOS) that was mainly concerned with three aspects (selection of patients, comparability of groups, and assessment of outcomes). Studies achieving more than seven points on the NOS were regarded to be of high quality.

Subgroups were generated if at least two studies were available; otherwise, subgroup analyses were not performed. A two-tailed P value < 0.05 was considered statistically significant.

### Outcomes of Comparison and Statistical Analysis

Short-term outcomes included duration of operation (min), blood loss during operation (mL), incidence of blood transfusion, prevalence rate of postoperative positive margins, postoperative (30 day) morbidity and mortality. Long-term outcomes included OS and DFS.

For dichotomous variables, odds ratio (OR, calculated by the numbers of events and patients) with a 95% confidence interval (CI) was used for analysis. For continuous variables, weighted mean difference (WMD) with a 95% CI was used for analysis. For studies describing such variables as median and range, corresponding mean and variance were estimated by the methods proposed by Hozo^28^. For comparison of OS and DFS, the hazard ratio (HR, describing a summary statistic for censored outcomes) with a 95% CI was used. An HR value (reference NAR group) less than one indicated a survival benefit favoring AR over NAR. If such survival-type data or additional key data were absent in the article, the corresponding author of each report was contacted by e-mail. In the absence of replies from the authors, the methods introduced by Tierney^29^ were used to calculate HR and corresponding CI from other information such as the OS Kaplan-Meier curve. For comparison of categorical variables, the chi-squared (χ^2^) test or Fisher’s exact test was utilized, as appropriate. A fixed effect model was used in the absence of significant heterogeneity (I^2^ <50%); otherwise, a random effect model was used. Review Manager (version 5.3.5) software was utilized to conduct the meta-analysis. Further statistical analysis of time to event data (HR and 95% CI) were performed by STATA (version 12.0) statistical software. Between-study heterogeneity was assed using Cochrane’s Q and I^2^ tests. Begg’s funnel plot and Egger’s tests were used to assess publication bias. Sensitivity analysis was performed using studies of high quality. Subgroup analysis was used to explore the between-study heterogeneity according to predefined parameters: cohorts without chemotherapy and sample size (size ≥200 and size <200).

## Results

### Study Selection and Patients Characteristics

**Figure 1:**
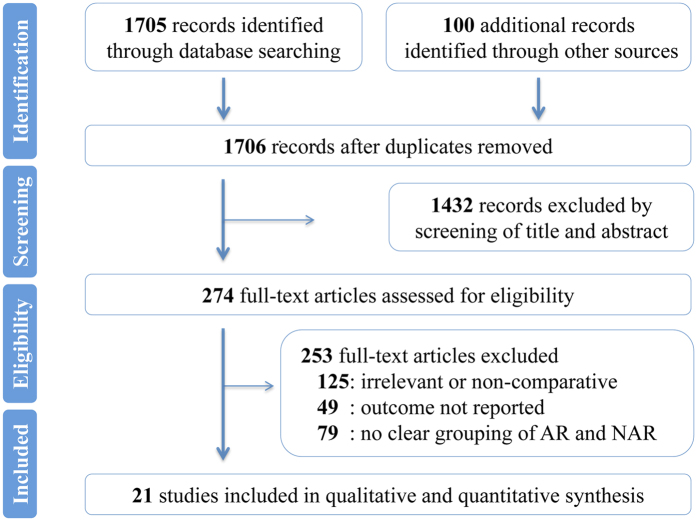
PRISMA flowchart of the study selection.

A PRISMA flowchart of the Study selection was shown in Fig. 1. The search returned a total of 1706 references. By meticulously screening titles and abstracts, 1432 references were eliminated. Among the remaining 274 potentially appropriate studies, 253 were excluded by full text analysis for matching one of the exclusion criteria. Ultimately, a total of 21 studies reporting on 5207 patients were eligible to be included in the present meta-analysis^19^,^20^,^21^,^22^,^23^,^24^,^25^,^30^,^31^,^32^,^33^,^34^,^35^,^36^,^37^,^38^,^39^,^40^,^41^,^42^,^43^. All 21 studies were retrospective, nonrandomized studies published between 1987 and 2016 that were conducted in the United States of America (five studies), Italy (four studies), Japan (four studies), the United Kingdom (two studies), Germany (two studies), Netherland (one study), Turkey (one study) or Sweden (one study) or that were multicenter (one study). Among the 5207 patients enrolled, 3034 (58.3%) underwent AR procedure and 2173 (41.7%) underwent NAR procedure. The sample size for these studies varied from 31 to 1001. The median follow-up length of the studies ranged from 20 to 94 months. For most of the studies, the mean or median age was in the 60 s or 70 s. The proportions of male patients were similar in both groups. Besides, based on the available data of some studies, the proportions of patients with carcinoembryonic antigen (CEA) level less than 200 ng/mL and amounts of tumors were comparable in both groups. The site of the primary tumor was reported for 1601 neoplasms, of which 1117 (69.8%) were located in colon and 484 (30.2%) in rectum, respectively. In AR group, 612 of the total 850 (72.0%) neoplasms were located in the colon; in NAR group, 505 of the total 751 (67.2%) neoplasms were in the colon. In addition, a commensurate rate of patients who received chemotherapy was observed between AR group and NAR group (χ^2^ test, P = 0.58). The number of patients with bilobar metastases in the liver were comparable in the AR and NAR groups (22.95% versus 20.37%, P = 0.15). Similarly, no significant differences between the two groups were identified in terms of the presence of resectable extrahepatic metastases (2.29% in AR group versus 1.27% in NAR group, P = 0.09). Study characteristics, patient demographic information, and quality scoring were summarized in Table 1. Main outcomes were outlined in Table 2.

### Short-term Outcome

Twelve of the 21 studies described the short-term outcomes (operative and postoperative)^21^,^23^,^24^,^25^,^30^,^31^,^32^,^33^,^34^,^36^,^41^,^42^.

**Figure 2:**
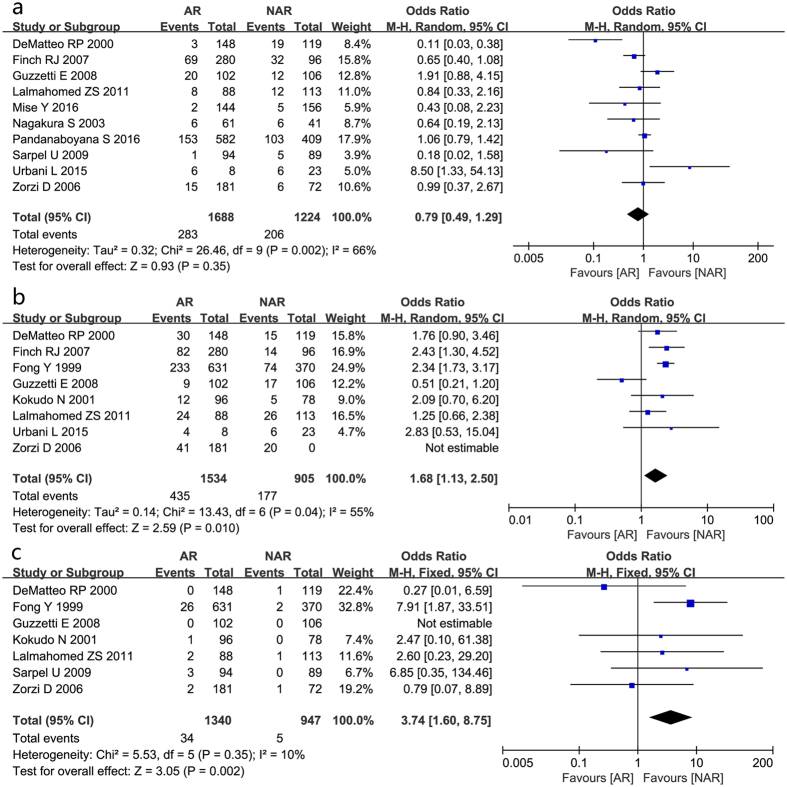
Results of the meta-analysis on short-term outcome. (postoperative positive margins (**a**), postoperative morbidity (**b**) and mortality (**c**)).

Ten studies were pooled to find a combined effect on the prevalence rate of postoperative positive margins. The results showed an OR of 0.79 in favor of AR group; however, the 95% CI crossed the no-effect line (95% CI 0.49–1.29, Fig. 2a).

The analysis of blood loss during operation revealed no significant differences. As to incidence of blood transfusion, the combined results favored NAR group (OR 2.94, 95% CI 1.87–4.62).

With regard to duration of operation, overall outcome from four studies indicated that NAR group was characterized by a reduced duration of operation in comparison with AR group (WMD 43.62, 95% CI 5.25–81.99).

Postoperative morbidity was reported in eight studies involving 2439 patients. The difference identified by the pooled analysis approached statistical significance in favor of NAR group (OR 1.68, 95% CI 1.13–2.50, Fig. 2b), with moderate between-study heterogeneity.

A similar advantage of mortality favoring NAR group was also presented among seven studies with slight between-study heterogeneity, indicating that NAR was associated with a lower mortality rate than AR (OR 3.74, 95% CI 1.60–8.75, Fig. 2c).

### Long-term Outcome

**Figure 3:**
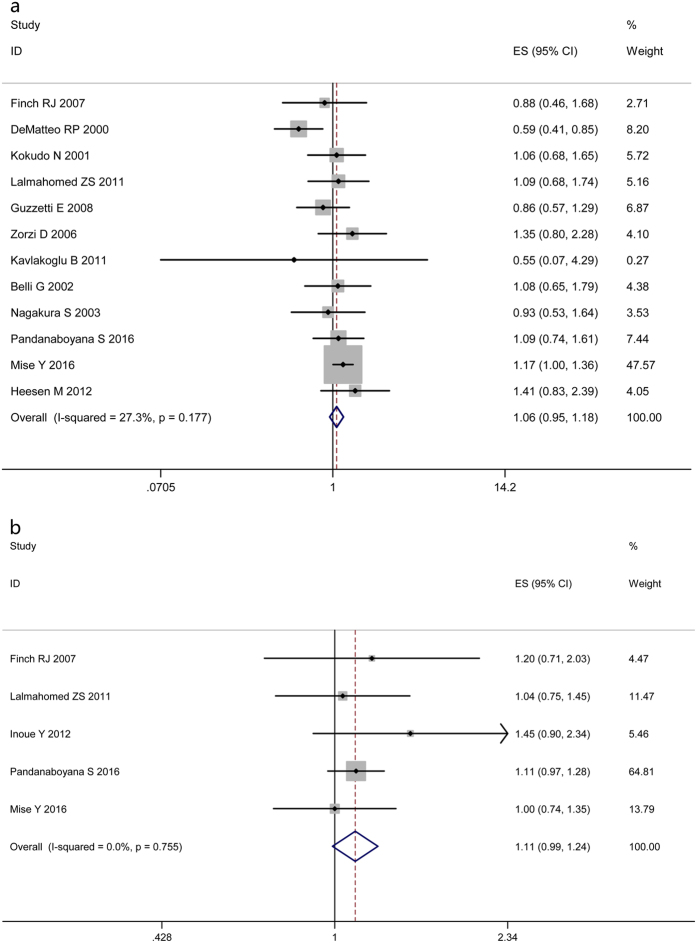
Results of the meta-analysis on OS (**a**) and DFS (**b**).

Thirteen of the 21 studies described the long-term outcomes (OS and DFS). For OS, HR values extracted from 12 studies assessing 1803 patients were put into overall analysis^21^,^23^,^24^,^25^,^31^,^32^,^35^,^36^,^40^,^41^,^42^. No clear evidence of a benefit of AR on time to survival was identified (HR 1.06, 95% CI 0.95–1.18, Fig. 3a). With regard to DFS, pooling the data from five studies showed no significant difference between the two groups (HR 1.11, 95% CI 0.99–1.24, Fig. 3b)^23^,^31^,^39^,^41^,^42^.

### Subgroup Analyses

**Figure 4:**
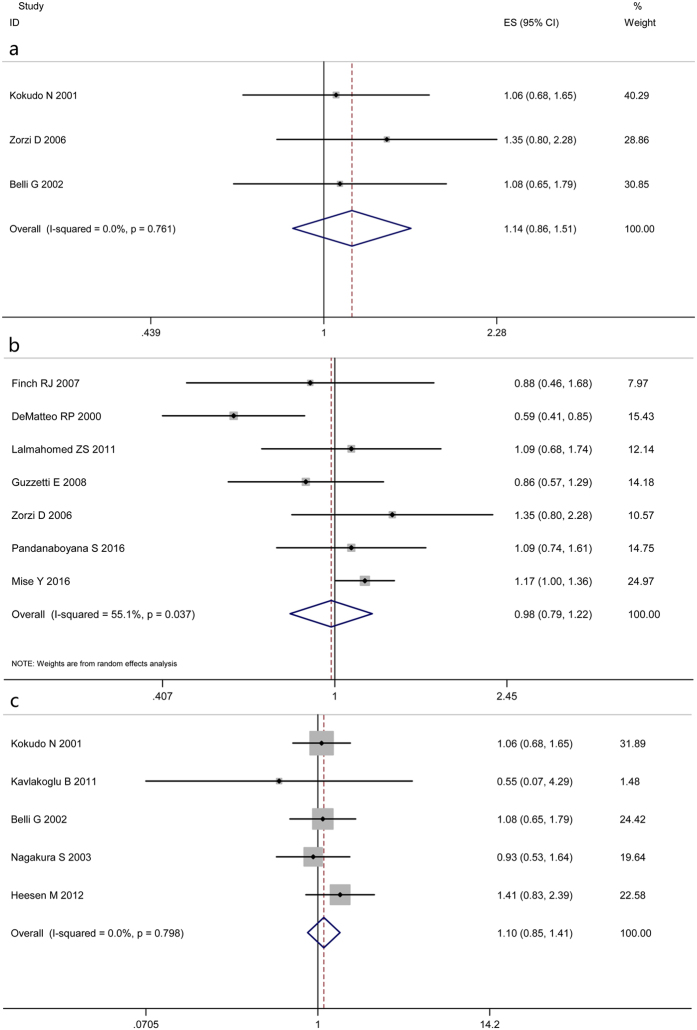
Results of three subgroup analyses of OS. (subgroup of cohorts entirely without chemotherapy (**a**), subgroup of sample size ≥200 (**b**) and size <200 (**c**)).

In accordance with the predefined parameters, namely, the subgroup of cohorts entirely without chemotherapy, subgroup of sample size ≥200 and size <200, three subgroup analyses of OS were conducted. Uniformly, pooled analyses showed similar results in comparison with the overall finding (Fig. 4). For the limited studies for inclusion, subgroup analyses of DFS were not performed. All the above results are detailed in Table 2.

### Analysis of Sensitivity and Test for Publication Bias

**Figure 5:**
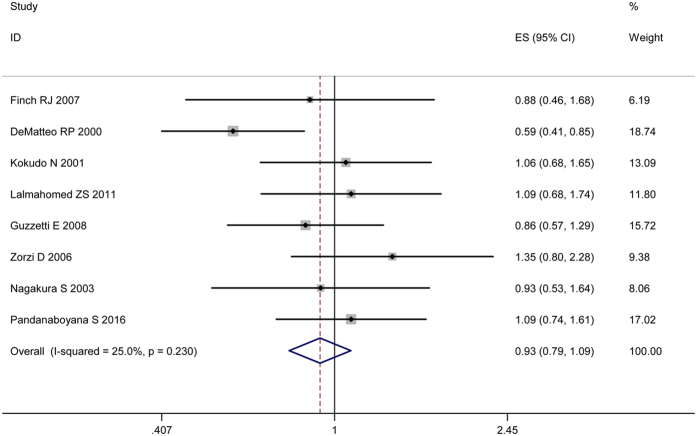
Results of the sensitivity analysis basing on nine high-quality studies.

**Figure 6:**
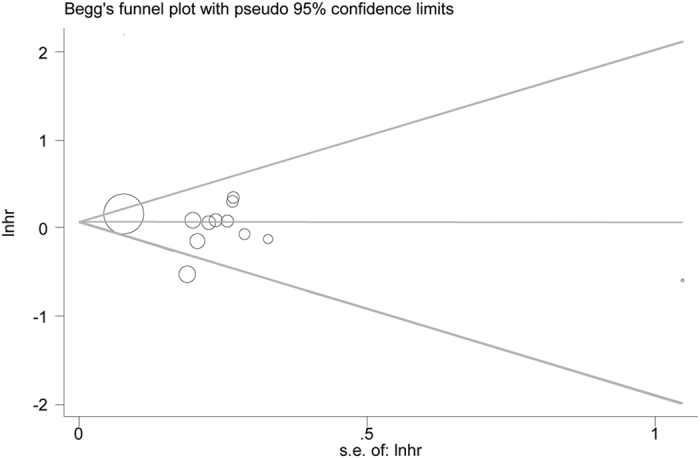
Begg’s funnel plot to evaluate OS.

Basing on nine high-quality studies^21^,^23^,^24^,^25^,^30^,^31^,^32^,^34^,^35^,^36^,^39^,^41^, a further sensitivity analysis was performed. No significant changes of the previous outcomes were produced in comparison with the overall analysis. As regards to OS, a commensurate result (HR 0.93, 95% CI 0.79–1.09, Fig. 5) was produced. And the between-study heterogeneities of the previous comparisons were slightly reduced. There was no evident publication bias based on Egger’s test (P = 0.32), with symmetry in Begg’s funnel plot (Fig. 6).

## Discussion

This meta-analysis has broadly reviewed the differences of a variety of patient important outcomes between AR and NAR in performing hepatectomy for CRLM. The results suggest that AR is inferior to NAR in terms of duration of operation, as well as incidence of postoperative morbidity and mortality. Prevalence rate of postoperative positive margins and blood loss were comparable between AR and NAR. Regarding long-term outcomes, OS and DFS did not differ significantly between AR and NAR. These findings are commensurate with the sensitivity analysis of high-quality studies.

Hepatectomy for secondary liver malignancies remains to be a crucial and useful therapeutic option. Widely recognized advantages in OS and DFS were achieved by AR in surgical treatment of HCC compared to NAR^10^,^11^,^12^,^13^,^15^,^16^,^17^. However, such benefits of AR for CRLM were not demonstrated in comparison with NAR in the present meta-analysis, with both groups showing equivalent results in terms of long-term survival outcomes (OS and DFS).

Such inconsistent results may be explained from the following two aspects: the influences of postoperative margins and the different disseminating modes of tumor cells. (1) Postoperative margin largely depending upon the surgical technique has been extensively investigated and consistently considered to be strongly associated with OS and DFS in CRLM. Consensus has been widely accepted that a positive surgical margin is a powerful predictor of patient survival and recurrence^7^,^34^,^44^. As has been reported, the rate for five-year survival ranges only from 17.1% to 20% for patients with positive margins compared with that ranging from 37% to 63.8% with negative margins^7^,^34^. As to median survival, the median length was 23 months for patients with positive margins, less than 45 months with negative margins^34^. Besides, overall recurrence rates were significantly different between patients with positive margins and with negative margins (51.1% and 38.6%, respectively)^34^. Furthermore, both univariate and multivariate analysis revealed that a positive resection margin predicted an increased recurrence rate (relative risk (RR) 2.60, 95% CI 1.55–4.38 and RR 2.34, 95% CI 1.37–4.01, respectively)^44^. In the present meta-analysis, there were no statistically significant differences in the prevalence rate of positive margins. The similarity in the prevalence of postoperative margin would consequently result in no significant difference of OS and DFS between AR and NAR. Therefore, our study confirms the correlation between margin status and its impact on OS and DFS. (2) As to tumor disseminating mode, secondary malignancies of liver may run some particular metastatic modes^18^,^45^,^46^,^47^ involving trans-arterial spread to the liver from various sources (pulmonary metastasis, the other metastasis, recurrent foci) and intrahepatic spread via changed portal venous circulation, which differs greatly from that of primary liver malignances. In theory, HCC tumor cells originating from the liver itself are thought to spread through the intra-segmental portal vein in the same segment. By erasing the tumor-bearing intra-segmental portal vein together with the corresponding segment, AR may effectively remove the intra-segmental metastasis and thus achieving a favorable outcome for HCC patients. Tumor cells of CRLM derived from the colon or rectum lesions travel through the superior mesenteric vein (SMV) and proximal portal vein and then flow into the liver^18^. The blood-borne metastasis is likely to be delivered evenly to any part of the liver. Previous reports^46^,^48^,^49^ using autopsy showed that colorectal carcinoma metastasis were distributed homogeneously in hepatic parenchyma. Shirai Y.^18^ described the distribution of a total of 67 liver metastases from the left colon, of which 28 were in the right lobe, 16 in the left lobe and 23 in both lobes. Besides, these findings, to some degree, corroborate the notion that the prevalence of extrahepatic metastasis for CRLM is more common than that for HCC. The above analysis may explain why AR did not provide a survival advantage over NAR for CRLM^18^,^50^.

At the meantime, the pooled analysis demonstrated that AR had a higher risk of post-operative morbidity (OR 1.68, 95% CI 1.13–2.50) and mortality (OR 3.74, 95% CI 1.60–8.75) than NAR. An increased morbidity rate could be due to a heightened surgical stress caused by the AR itself. As previously reported^31^,^51^,^52^,^53^, AR featuring higher level of surgical technique difficulty would often be associated with longer operation duration and more liver parenchyma loss. And this is consistent with the results from the studies included in this meta-analysis^21^,^24^,^31^,^33^. Bile leakage, wound infection and intra-abdominal collections constituting the major types of complications all show evident preferences to AR group over NAR group. Taken together, AR might promote the incidence of postoperative morbidity. The main cause for the inferiority of AR to NAR in terms of mortality is thought to be its larger loss of liver parenchyma. With more extensive parenchymal resection, AR would consequently carry a more substantial risk. As reported by Lalmahomed ZS^23^, postoperative hepatic failure resulting from insufficiency of liver remnant was the primary cause of mortality in AR group.

With regard to the longer operation duration of AR, it could be explained by the fact that standard AR is involved with some additional extensive surgical procedures, such as hepatic pedicle dissection or even segmental staining.

It was notable that the pooled result of blood loss during operation revealed no statistically significant differences. But a higher incidence of blood transfusion was found in AR (OR 2.94, 95% CI 1.87–4.62), which appeared not to agree well with earlier reports^12^,^54^,^55^ that concluded similar incidences of blood transfusion between AR and NAR. These inconsistent results could be caused by rather small sample sizes and selection bias as there are only three studies that assessed relevant data.

To our best knowledge, there is only one published meta-analysis concerning this topic^56^, due to the imperfectness in literature search and the neglect of variability or between-study heterogeneities, and some of its results remain inconclusive.

Comparatively speaking, our present study has three main strengths. (1) A substantial size of the studies included was produced by a comprehensive and extensive searching strategy. (2) As to time to event data, the best option of using HR value to perform the pooled analysis of OS effect was conducted. (3) On subgroup and sensitivity analyses, similar results were produced and thus confirmed the overall findings. Hence, our results were reliable and robust.

In spite of the above-mentioned improvements, certain limitations of the present study should be taken into consideration. The main limitation was that no RCTs were available to get included, thus reducing the reliability of the results. Besides, the lack of relevant data did not permit comprehensive subgroup analysis according to additional parameters, such as tumor size, tumor amount, primary tumor location, the use of chemotherapy, extent of metastases in the liver (unilobar or bilobar distribution of metastases) and the presence of resectable extrahepatic metastases (despite the fact that most of the parameters remained comparable between studies, as shown in Table 1), to be conducted. In addition, several HR values were calculated by corresponding OS Kaplan-Meier curves, because of the unavailability of these values in the articles and absence of replies from the authors. Finally, some of the between-study heterogeneities were relatively obvious, which might have been caused by the differences in sample size or other factors among these studies, and by the limited studies for inclusion. Hence random effect models were implemented for those comparisons. Nevertheless, the present analysis undoubtedly represents one more step in obtaining a more reliable and up-to-date evidence to give a relatively persuasive argument for resection type in CRLM.

To conclude, AR seems to have no prognostic advantages over NAR for the treatment of CRLM. Besides, AR is inferior to NAR with respect to incidence of postoperative morbidity and mortality. In addition there has been concern that the policy of AR would relatively restrict later surgical treatment possibilities for recurrent lesions. Taken together, our current results might not give support to AR for CRLM patients. Surgeons ought to be cautious when they select the procedures in the surgical treatment of CRLM. Further multicenter and high-quality RCTs will be required to support this conclusion.

## Additional Information

**How to cite this article**: Tang, H. *et al*. Comparison of Anatomical and Nonanatomical Hepatectomy for Colorectal Liver Metastasis: A Meta-Analysis of 5207 Patients. *Sci. Rep.* **6**, 32304; doi: 10.1038/srep32304 (2016).

## Figures and Tables

**Table 1:** Characteristics of included studies.

First author	Year	Country	No. of Patients			Gender (M/F)		Age		Primary tumor (Colon/Rectum)		
			Total	AR	NAR	AR	NAR	AR	NAR	AR	NAR	
Ekberg H	1987	Sweden	68	55	13	—	—	—	—	—	—	CONTINUED
Doci R	1991	Italy	95	46	49	—	—	—	—	—	—	
Scheele J	1995	Germany	350	291	59	—	—	—	—	—	—	
Wanebo HJ	1996	the US	74	51	23	—	—	—	—	—	—	
Fong Y	1999	the US	1001	631	370	—	—	—	—	—	—	
Yamamoto J	1999	Japan	96	23	73	—	—	—	—	—	—	
DeMatteo RP	2000	the US	267	148	119	60/88	67/52	<70 (n = 110)	<70 (n = 83)	110/38	86/33	
Kokudo N	2001	Japan	174	96	78	54/42	46/32	58.7 ± 1.0†	60.3 ± 1.2†	71/25	49/29	
Belli G	2002	Italy	181	56	125	—	—	—	—	—	—	
Nagakura S	2003	Japan	102	61	41	35/26	28/13	<60 (n = 27)	<60 (n = 11)	39/22	26/15	
Zorzi D	2006	Multicenter	253	181	72	113/68	46/26	<65 (n = 120)	<65 (n = 44)	113/46	42/17	
Finch RJ	2007	the UK	376	280	96	171/109	64/32	63 (26–84)¶	63 (24–79)¶	—	—	
Guzzetti E	2008	Italy	208	102	106	58/44	63/43	<70 (n = 78)	<70 (n = 77)	55/21	60/31	
Sarpel U	2009	the US	183	94	89	54/40	51/38	60.8 ± 10.4†	62.3 ± 11.6†	60/10	59/12	
Lalmahomed ZS	2011	Netherlands	201	88	113	56/22	70/43	65 (30–82)¶	65 (36–86)¶	55/33	59/54	
Kavlakoglu B	2011	Turkey	42	18	24	—	—	—	—	—	—	
Inoue Y	2012	Japan	106	32	74	—	—	—	—	—	—	
Heesen M	2012	Germany	108	47	61	—	—	—	—	—	—	
Urbani L	2015	Italy	31	8	23	5/3	13/10	64 (33–83)¶	68 (41–77)¶	4/4	11/12	
Pandanaboyana S	2016	the UK	991	582	409	—	—	<65 (n = 282)	<65 (n = 175)	—	—	
Mise Y	2016	the US	300	144	156	80/64	94/62	58 (22–87)¶	60 (30–88)¶	105/39	113/43	
**First author**	**CEA Level (ng/mL)**		**Tumor Amount**		**Tumor Presentation Syn/Meta**			**NOS**				
	**AR**	**NAR**	**AR**	**NAR**	**AR**	**NAR**	**Follow-up (months)**					
Ekberg H	—	—	—	—	—	—	20 (3–167)¶	5				
Doci R	—	—	—	—	—	—	17 (1–97)¶	6				
Scheele J	—	—	—	—	—	—	—	6				
Wanebo HJ	—	—	—	—	—	—	—	6				
Fong Y	—	—	—	—	—	—	32¶	7				
Yamamoto J	—	—	—	—	—	—	—	6				
DeMatteo RP	<200 (n = 101)	<200 (n = 84)	>1 (n = 30)	>1 (n = 23)	—	—	—	7				
Kokudo N	—	—	>1 (n = 42)	>1 (n = 36)	50/46	22/56	—	7				
Belli G	‒‒	—	—	—	—	—	—	6				
Nagakura S	<100 (n = 41)	<100 (n = 34)	>3 (n = 7)	>3 (n = 2)	—	—	94 (4–234)¶	8				
Zorzi D	<200 (n = 160)	<200 (n = 63)	>1 (n = 82)	>1 (n = 27)	73/108	25/47	25¶	8				
Finch RJ	18 (1–37140)¶	5 (1–12124)¶	2 (2–14)¶	1 (1–9)¶	117/163	36/60	33 (24–144)¶	8				
Guzzetti E	<200 (n = 52)	<200 (n = 51)	>1(n = 43)	>1 (n = 39)	—	—	—	8				
Sarpel U	—	—	1.7 ± 1.2†	1.4 ± 1.0†	—	—	34¶	7				
Lalmahomed ZS	<200 (n = 78)	<200 (n = 107)	2 (1–7)¶	1 (1–7)¶	35/53	43/70	35 (1–111)¶	8				
Kavlakoglu B	—	—	—	—			40.40 ± 12.87†	6				
Inoue Y	—	—	1.8 ± 1.4†	2.2 ± 2.2†	—	—	—	6				
Heesen M	—	—	—	—	—	—	31.7 ± 1.6†	6				
Urbani L	—	—	2 (1–10)¶	1 (1–12)¶	6/2	15/8	25.2 (0.3–62.7)¶	7				
Pandanaboyana S	—	—	2 ± 1.5†	2 ± 1.5†	294/288	228/181	32.2 (17.5–56.9)¶	7				
Mise Y	2.9 (0.4–250.3)¶	2.5 (0.4–430.9)¶	—	—	—	—	37 (2–208)¶	6				

AR: Anatomical resection; NAR: Nonanatomical resection; Syn: synchronous metastasis (within 6 months of resection of the primary tumor); Meta: metachronous metastasis (after 6 months of resection of the primary tumor); NOS: Newcastle-Ottawa Scale.

^¶^Values are median with or without range.

^†^Values are mean and standard deviation.

**Table 2:** Results of meta-analysis comparing AR and NAR for CRLM.

Outcome		Number of studies	Number of patients			HR/OR/WMD (95%) CI	P value	I^2^ (%)	Effect model
					AR					NAR	Total
**Long-term**									
OS	Overall	12	1803	1400	3203	1.06 (0.95–1.18)¶	0.18	27.30	Fixed
	Without Chemotherapy	3	608	333	275	1.14 (0.86–1.51)¶	0.76	0	Fixed
	Size ≥200	7	1525	1071	2596	0.98 (0.79–1.22)¶	0.80	0	Fixed
	Szie <200	5	278	329	607	1.10 (0.85–1.41)¶	0.04	55.10	Random
**DFS**		5	1126	848	1974	1.11 (0.99–1.24)¶	0.76	0	Random
**Short-term**									
Postoperative margin		10	1688	1224	2912	0.79 (0.49–1.29)+	0.35	66.00	Random
Blood loss (mL)		4	490	459	949	243.52 (−78.29–565.33)†	0.14	99.00	Random
Blood transfusion		3	190	225	415	2.94 (1.87–4.62)+	<0·01	0	Fixed
Duration of operation (min)		4	354	326	680	43.62 (5.25–81.99)†	0.03	99.00	Random
30-day morbidity		8	1534	905	2439	1.68 (1.13–2.50)+	0.01	55.00	Random
30-day mortality		7	1340	947	2287	3.74 (1.60–8.75)+	<0·01	10.00	Fixed

AR: Anatomical resection; NAR: Nonanatomical resection; HR: hazard ratio; OR: odds ratio; WMD: weighted mean difference; CI: confidence interval; OS: overall survival; DFS: disease free survival.

^¶^Values are HR with corresponding CI.

^+^Values are OR with corresponding CI.

^†^Values are WMD with corresponding CI; Statistically significant results are shown in bold.

## References

[b1] Jemal A. . Global cancer statistics. CA Cancer J Clin 61, 69–90 (2011). 21296855 10.3322/caac.20107

[b2] Manfredi S. . Epidemiology and management of liver metastases from colorectal cancer. Ann. Surg. 244, 254–259 (2006). 16858188 10.1097/01.sla.0000217629.94941.cf PMC1602156

[b3] Cummings L. C., Payes J. D. & Cooper G. S. Survival after hepatic resection in metastatic colorectal cancer: a population-based study. Cancer 109, 718–726 (2007). 17238180 10.1002/cncr.22448

[b4] Leporrier J. . A population-based study of the incidence, management and prognosis of hepatic metastases from colorectal cancer. Br J Surg 93, 465–474 (2006). 16523446 10.1002/bjs.5278

[b5] Xu J. . Hepatectomy for liver metastasis of colorectal cancer. Int J Colorectal Dis 24, 419–425 (2009). 19096855 10.1007/s00384-008-0619-5

[b6] Ueno H., Mochizuki H., Hatsuse K., Hase K. & Yamamoto T. Indicators for treatment strategies of colorectal liver metastases. Ann. Surg. 231, 59–66 (2000). 10636103 10.1097/00000658-200001000-00009 PMC1420966

[b7] Pawlik T. M. . Effect of surgical margin status on survival and site of recurrence after hepatic resection for colorectal metastases. Ann. Surg. 241, 715–722, discussion 722–724 (2005). 15849507 10.1097/01.sla.0000160703.75808.7d PMC1357126

[b8] Abdalla E. K. . Recurrence and outcomes following hepatic resection, radiofrequency ablation, and combined resection/ablation for colorectal liver metastases. Ann. Surg. 239, 818–825, discussion 825–827 (2004). 15166961 10.1097/01.sla.0000128305.90650.71 PMC1356290

[b9] Makuuchi M., Hasegawa H. & Yamazaki S. Ultrasonically guided subsegmentectomy. Surgery, gynecology & obstetrics 161, 346–350 (1985). 2996162

[b10] Chen J. . Survival after anatomic resection versus nonanatomic resection for hepatocellular carcinoma: a meta-analysis. Dig. Dis. Sci. 56, 1626–1633 (2011). 21082347 10.1007/s10620-010-1482-0

[b11] Yamashita Y. . Longterm favorable results of limited hepatic resections for patients with hepatocellular carcinoma: 20 years of experience. J. Am. Coll. Surg. 205, 19–26 (2007). 17617328 10.1016/j.jamcollsurg.2007.01.069

[b12] Hasegawa K. . Prognostic impact of anatomic resection for hepatocellular carcinoma. Ann. Surg. 242, 252–259 (2005). 16041216 10.1097/01.sla.0000171307.37401.db PMC1357731

[b13] Kamiyama T. . The impact of anatomical resection for hepatocellular carcinoma that meets the Milan criteria. J Surg Oncol 101, 54–60 (2010). 19798687 10.1002/jso.21414

[b14] Lu W. P. & Dong J. H. Hepatectomy for hepatocellular carcinoma in the era of liver transplantation. World J. Gastroenterol. 20, 9237–9244 (2014). 25071316 10.3748/wjg.v20.i28.9237 PMC4110553

[b15] Ye J. Z. . Recurrence after anatomic resection versus nonanatomic resection for hepatocellular carcinoma: a meta-analysis. Asian Pac. J. Cancer Prev. 13, 1771–1777 (2012). 22901120 10.7314/apjcp.2012.13.5.1771

[b16] Zhou Y., Xu D., Wu L. & Li B. Meta-analysis of anatomic resection versus nonanatomic resection for hepatocellular carcinoma. Langenbecks Arch Surg 396, 1109–1117 (2011). 21476060 10.1007/s00423-011-0784-9

[b17] Cucchetti A. . A comprehensive meta-regression analysis on outcome of anatomic resection versus nonanatomic resection for hepatocellular carcinoma. Ann. Surg. Oncol. 19, 3697–3705 (2012). 22722807 10.1245/s10434-012-2450-z

[b18] Shirai Y. . Colorectal carcinoma metastases to the liver. Does primary tumor location affect its lobar distribution. Cancer 77, 2213–2216 (1996). 8635086 10.1002/(SICI)1097-0142(19960601)77:11<2213::AID-CNCR5>3.0.CO;2-Q

[b19] Yamamoto J. . Factors influencing survival of patients undergoing hepatectomy for colorectal metastases. Br J Surg 86, 332–337 (1999). 10201774 10.1046/j.1365-2168.1999.01030.x

[b20] Wanebo H. J., Chu Q. D., Vezeridis M. P. & Soderberg C. Patient selection for hepatic resection of colorectal metastases. Arch Surg 131, 322–329 (1996). 8611099 10.1001/archsurg.1996.01430150100019

[b21] DeMatteo R. P. . Anatomic segmental hepatic resection is superior to wedge resection as an oncologic operation for colorectal liver metastases. J. Gastrointest. Surg. 4, 178–184 (2000). 10675241 10.1016/s1091-255x(00)80054-2

[b22] Scheele J., Stang R., Altendorf-Hofmann A. & Paul M. Resection of colorectal liver metastases. World J Surg 19, 59–71 (1995). 7740812 10.1007/BF00316981

[b23] Lalmahomed Z. S. . Anatomical versus nonanatomical resection of colorectal liver metastases: is there a difference in surgical and oncological outcome. World J Surg 35, 656–661 (2011). 21161655 10.1007/s00268-010-0890-9 PMC3032901

[b24] Kokudo N. . Anatomical major resection versus nonanatomical limited resection for liver metastases from colorectal carcinoma. Am. J. Surg. 181, 153–159 (2001). 11425058 10.1016/s0002-9610(00)00560-2

[b25] Zorzi D. . Comparison between hepatic wedge resection and anatomic resection for colorectal liver metastases. J. Gastrointest. Surg. 10, 86–94 (2006). 16368496 10.1016/j.gassur.2005.07.022

[b26] Liberati A. . The PRISMA statement for reporting systematic reviews and meta-analyses of studies that evaluate health care interventions: explanation and elaboration. J Clin Epidemiol 62, e1–34 (2009). 19631507 10.1016/j.jclinepi.2009.06.006

[b27] Moher D., Liberati A., Tetzlaff J. & Altman D. G. Preferred reporting items for systematic reviews and meta-analyses: the PRISMA Statement. Open Med 3, e123–e130 (2009). 21603045 PMC3090117

[b28] Hozo S. P., Djulbegovic B. & Hozo I. Estimating the mean and variance from the median, range, and the size of a sample. BMC Med Res Methodol 5, 13 (2005). 15840177 10.1186/1471-2288-5-13 PMC1097734

[b29] Tierney J. F., Stewart L. A., Ghersi D., Burdett S. & Sydes M. R. Practical methods for incorporating summary time-to-event data into meta-analysis. Trials 8, 16 (2007). 17555582 10.1186/1745-6215-8-16 PMC1920534

[b30] Sarpel U. . Does anatomic versus nonanatomic resection affect recurrence and survival in patients undergoing surgery for colorectal liver metastasis. Ann. Surg. Oncol. 16, 379–384 (2009). 19020941 10.1245/s10434-008-0218-2

[b31] Finch R. J. . Effect of type of resection on outcome of hepatic resection for colorectal metastases. Br J Surg 94, 1242–1248 (2007). 17657718 10.1002/bjs.5640

[b32] Guzzetti E. . Impact of type of liver resection on the outcome of colorectal liver metastases: a case-matched analysis. J Surg Oncol 97, 503–507 (2008). 18425789 10.1002/jso.20979

[b33] Urbani L. . Minor-but-Complex Liver Resection: An Alternative to Major Resections for Colorectal Liver Metastases Involving the Hepato-Caval Confluence. Medicine (Baltimore) 94, e1188 (2015). 26200628 10.1097/MD.0000000000001188 PMC4603012

[b34] Fong Y., Fortner J., Sun R. L., Brennan M. F. & Blumgart L. H. Clinical score for predicting recurrence after hepatic resection for metastatic colorectal cancer: analysis of 1001 consecutive cases. Ann. Surg. 230, 309–318, discussion 318–321 (1999). 10493478 10.1097/00000658-199909000-00004 PMC1420876

[b35] Belli G. . Liver resection for hepatic metastases: 15 years of experience. J Hepatobiliary Pancreat Surg 9, 607–613 (2002). 12541048 10.1007/s005340200082

[b36] Nagakura S. . Major hepatic resection reduces the probability of intrahepatic recurrences following resection of colorectal carcinoma liver metastases. Hepatogastroenterology 50, 779–783 (2003). 12828084

[b37] Doci R. . One hundred patients with hepatic metastases from colorectal cancer treated by resection: analysis of prognostic determinants. Br J Surg 78, 797–801 (1991). 1873704 10.1002/bjs.1800780711

[b38] Ekberg H. . Pattern of recurrence in liver resection for colorectal secondaries. World J Surg 11, 541–547 (1987). 3630198 10.1007/BF01655821

[b39] Inoue Y. . Resection margin with anatomic or nonanatomic hepatectomy for liver metastasis from colorectal cancer. J. Gastrointest. Surg. 16, 1171–1180 (2012). 22370732 10.1007/s11605-012-1840-7

[b40] Kavlakoglu B. . Surgical treatment of liver metastases from colorectal cancer: experience of a single institution. Arch Iran Med 14, 120–125 (2011). 21361719

[b41] Pandanaboyana S. . Impact of parenchymal preserving surgery on survival and recurrence after liver resection for colorectal liver metastasis. ANZ J Surg (2016). 10.1111/ans.13588 27111217

[b42] Mise Y. . Parenchymal-sparing Hepatectomy in Colorectal Liver Metastasis Improves Salvageability and Survival. Ann. Surg. 263, 146–152 (2016). 25775068 10.1097/SLA.0000000000001194

[b43] von H. M. . Parenchyma-preserving hepatic resection for colorectal liver metastases. Langenbecks Arch Surg 397, 383–395 (2012). 22089696 10.1007/s00423-011-0872-x

[b44] Gayowski T. J. . Experience in hepatic resection for metastatic colorectal cancer: analysis of clinical and pathologic risk factors. Surgery 116, 703–710, discussion 710–711 (1994). 7940169 PMC2967179

[b45] Leen E. . Hepatic perfusion changes in patients with liver metastases: comparison with those patients with cirrhosis. Gut 34, 554–557 (1993). 8491406 10.1136/gut.34.4.554 PMC1374320

[b46] Kishimoto R. . Segmental reversal of intrahepatic portal flow due to a liver metastasis. Br J Radiol 65, 1035–1038 (1992). 1450819 10.1259/0007-1285-65-779-1035

[b47] Utsunomiya T. & Matsumata T. Metastatic carcinoma in the cirrhotic liver. Am. J. Surg. 166, 776 (1993). 8273867 10.1016/s0002-9610(05)80698-1

[b48] Schulz W., Hagen C. & Hort W. The distribution of liver metastases from colonic cancer. A quantitative postmortem study. Virchows Archiv. A, Pathological anatomy and histopathology 406, 279–284 (1985). 3923704 10.1007/BF00704297

[b49] Strohmeyer T. & Schultz W. The distribution of metastases of different primary tumors in the liver. Liver 6, 184–187 (1986). 3747745 10.1111/j.1600-0676.1986.tb00287.x

[b50] Dionne L. The pattern of blood-borne metastasis from carcinoma of rectum. Cancer 18, 775–781 (1965). 14297474 10.1002/1097-0142(196506)18:6<775::aid-cncr2820180615>3.0.co;2-v

[b51] Bolton J. S. & Fuhrman G. M. Survival after resection of multiple bilobar hepatic metastases from colorectal carcinoma. Ann. Surg. 231, 743–751 (2000). 10767796 10.1097/00000658-200005000-00015 PMC1421062

[b52] Melendez J. . Extended hepatic resection: a 6-year retrospective study of risk factors for perioperative mortality. J. Am. Coll. Surg. 192, 47–53 (2001). 11192922 10.1016/s1072-7515(00)00745-6

[b53] Stewart G. D. . The extent of resection influences outcome following hepatectomy for colorectal liver metastases. Eur J Surg Oncol 30, 370–376 (2004). 15063889 10.1016/j.ejso.2004.01.011

[b54] Kaibori M. . Comparison of limited and anatomic hepatic resection for hepatocellular carcinoma with hepatitis C. Surgery 139, 385–394 (2006). 16546504 10.1016/j.surg.2005.08.035

[b55] Tanaka K. . Anatomic versus limited nonanatomic resection for solitary hepatocellular carcinoma. Surgery 143, 607–615 (2008). 18436008 10.1016/j.surg.2008.01.006

[b56] Sui C. J. . Anatomical versus nonanatomical resection of colorectal liver metastases: a meta-analysis. Int J Colorectal Dis 27, 939–946 (2012). 22215149 10.1007/s00384-011-1403-5

